# Supplementary material for: Broad and colossal edge supercurrent in Dirac semimetal Cd3As2 Josephson junctions
Source: Nat Commun. 2023 Oct 3;14:6162. doi: 10.1038/s41467-023-41815-4 (PMC10547728; doi:10.1038/s41467-023-41815-4)
Supplement: Supplementary file 1 — Supplementary Information [file 41467_2023_41815_MOESM1_ESM.pdf]

Supplementary Information for

Broad and colossal edge supercurrent in Dirac semimetal Cd_3As_2 Josephson junctions

Chun-Guang Chu[1], Jing-Jing Chen[2,3], An-Qi Wang[1]*, Zhen-Bing Tan[2,3]*, Cai-Zhen Li[2,3], Chuan Li[4], Alexander Brinkman[4], Peng-Zhan Xiang[1], Na Li[1], Zhen-Cun Pan[1], Hai-Zhou Lu[2], Dapeng Yu[2,3,5] and Zhi-Min Liao[1,5]*

[1] State Key Laboratory for Mesoscopic Physics and Frontiers Science Center for Nano-optoelectronics, School of Physics, Peking University, Beijing 100871, China

[2] Shenzhen Institute for Quantum Science and Engineering, Department of Physics, Southern University of Science and Technology, Shenzhen, 518055, China

[3] International Quantum Academy, Shenzhen 518048, China

[4] MESA+ Institute for Nanotechnology, University of Twente, 7500 AE Enschede, The Netherlands

[5]Hefei National Laboratory, Hefei 230088, China.

E-mail: anqi0112@pku.edu.cn; tanzb@sustech.edu.cn; liaozm@pku.edu.cn

This file contains supplementary Table 1, Figures 1-4, and Notes 1, 2.

Note 1: Derivation of the width and value of the edge supercurrent.

Note 2: Discussion on the origin of large broadening edge channels.

Supplementary Table 1 | The parameters of the measured junctions.

Junction number	Junction-1	Junction-2
L (nm)	800	600
W (μm)	4.8	4.4
I_c (μA) at 10 mK at V_g = 0 V	2	2.2

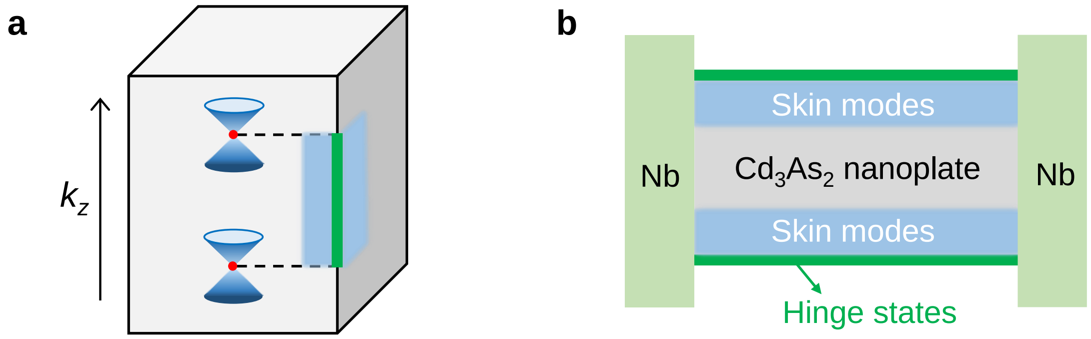

Supplementary Figure 1 | Hinge states and skin modes in a non-Hermitian higher-order Dirac semimetal.

a, Schematic of the hinge states and skin modes in momentum space. The hinge states (green line) connect the projection of bulk Dirac points on the hinge along the (001) direction. Skin modes arising from the non-Hermitian skin effect are denoted by light blue. For simplicity, only one hinge is demonstrated with boundary modes.

b, Spatial distribution of hinge states and skin modes in the Cd_3As_2 nanoplate device.

The Cd_3As_2 nanoplates are believed to possess three-dimensional bulk states, two-dimensional surface states, one-dimensional hinge states and additional non-Hermitian skin modes. The hinge states are due to the higher-order topological nature of Cd_3As_2, which are exhibited as segments connecting the projection of the bulk Dirac points along the hinges (Supplementary Fig. 1). Skin modes, as the consequence of the non-Hermitian open system, are also expected to exist in Cd_3As_2. These different electronic states have different superconducting coherence lengths, allowing one to distinguish them by increasing the channel length of the Josephson junction. Especially, for a Josephson junction with a long channel length, the bulk and surface supercurrents are significantly suppressed, and the hinge-state and skin-mode contributions become manifest and can be experimentally captured.

Below we compare the superconducting coherence length of these conduction channels. For a ballistic Josephson junction, the coherence length $\xi_0 = \hbar v_f / \Delta_0$ is about 760 nm by considering the Fermi velocity $v_f \sim 3 \times 10^5$ m/s and proximity-induced gap $\Delta_0 \sim 0.26$ meV of junction 1. While for a diffusive junction, the coherence length $\xi = \sqrt{\xi_0 l_e / D}$, where $D = 1, 2, 3$ is the transport dimension. Using the bulk mean free path $l_e^{bulk} = v_f \left(\frac{m^* \mu_e}{e}\right) = 26$ nm from the measured transfer curve

(Supplementary Fig. 3d), we can derive the bulk coherence length $\xi^{bulk} = 81$ nm. For 2D surface states, $\xi^{surface} = 760$ nm is obtained by using $l_e^{surface} \sim 1$ μm (ref. 1). Compared to the surface states, the quasi-1D channels (mixture of hinge states and skin modes) undergo fewer back scatterings and experience less coupling with bulk states, thus harboring a longer mean free path and coherence length. For junction 1 with a channel length of 800 nm, the bulk and surface carried supercurrents are greatly suppressed, giving rise to the supercurrent dominated by quasi-1D channels located at the sample edges and SQUID-like interference pattern.

The Josephson interferometry experiment provides an effective tool to capture the skin modes. Under an out-of-plane perpendicular magnetic field, the supercurrent interference effect leads to the specific $I_c(B_z)$ pattern, from which we can determine the spatial distribution of supercurrent via the Dynes and Fulton method. For junction 1, the extracted edge channel width is found to be much larger than that of hinge states (strongly confined to sample edges), indicating the contribution of skin modes.

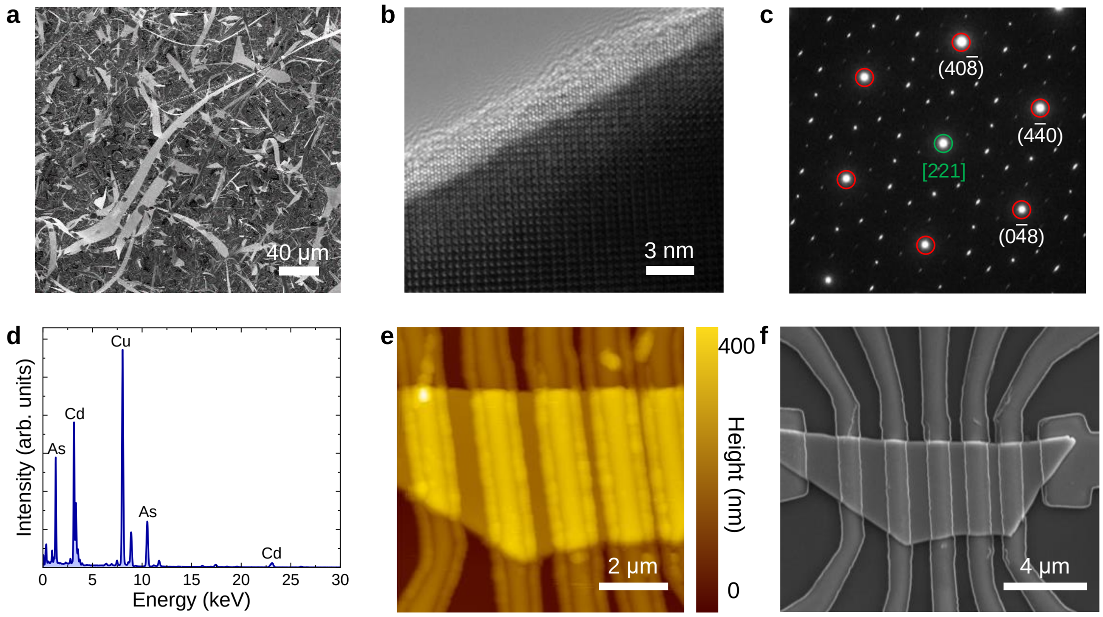

Supplementary Figure 2 | Characterization of the synthesized Cd_3As_2 nanoplates and the fabricated Josephson junctions.

a, Scanning electron microscopy (SEM) image of the as-grown nanoplates.

b, High-resolution transmission electron microscopy (TEM) image of a typical nanoplate with a 3 nm-thick oxide layer.

c, Corresponding selected area electron diffraction (SAED) pattern clearly shows the [221] zone axis, indicating the naturally grown surface is (112) crystal plane.

d, The energy-dispersive X-ray spectroscopy (EDS) spectrum of the nanoplate. Chemical composition analysis shows that the atomic ratio of Cd and As is approximately 3:2.

e, Atomic force microscope (AFM) image of the fabricated devices. The thickness of the Cd_3As_2 nanoplate is about 170 nm.

f, SEM image of the fabricated devices.

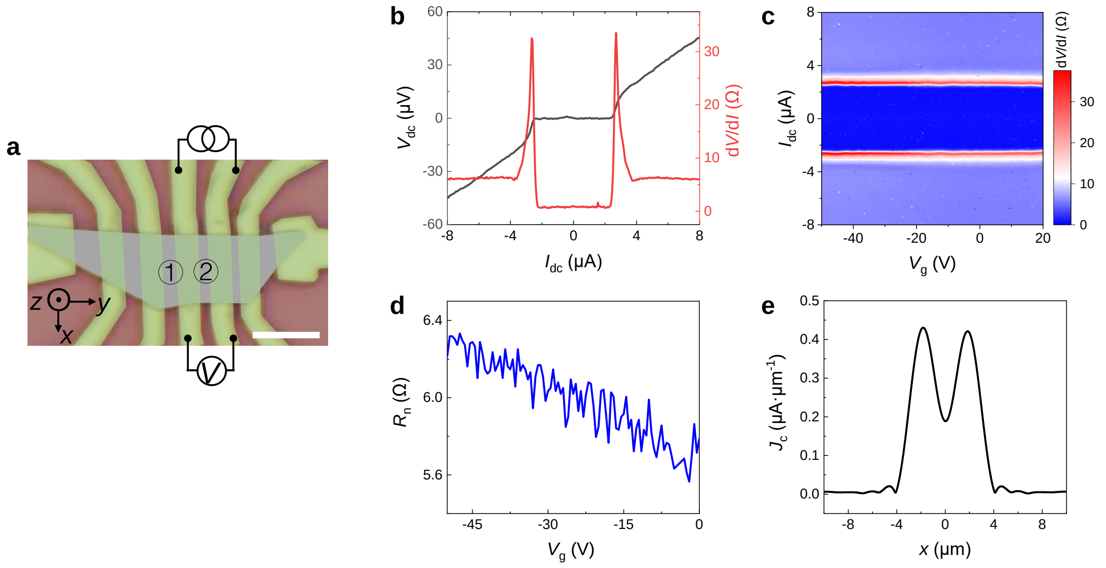

Supplementary Figure 3 | Characterization of the Cd_3As_2 nanoplate junction 2 with $L = 600$ nm.

a, Sketch of the device structure and measurement geometry for junction 2. Scale bar, 4 μm.

b, The dc voltage V_{dc} and differential resistance $\mathrm{d}V/\mathrm{d}I$ as a function of bias current I_{dc}. The bias I_{dc} is swept from negative to positive values. Below the critical current $I_c = 2.2$ μA, the junction is in the superconducting state.

c, Color map of $\mathrm{d}V/\mathrm{d}I$ as a function of I_{dc} and gate voltage V_g. The dark blue region represents the superconducting states of the junction. The upper boundary denotes the critical current, which is almost independent of V_g.

d, Normal-state resistance R_n versus the gate voltage V_g. R_n keeps increasing upon sweeping V_g to negative values, indicating the nanoplate is heavily electron-doped and the Fermi level cannot be modulated to the Dirac point even at $V_g = -50$ V.

e, The extracted supercurrent density $J_c(x)$ using the Dynes and Fulton method.

Supplementary Figure 3 shows the transport results measured on junction 2 with channel length $L = 600$ nm. The measurement geometry is depicted in Supplementary Fig. 3a. This junction has a critical current of about 2.2 μA, which is almost unchanged when tuning the gate voltage V_g (Supplementary Fig. 3b,c). We find the studied Cd_3As_2 nanoplate is heavily electron-doped and the electron density can be estimated as $n \sim 2 \times 10^{18}\ \mathrm{cm}^{-3}$ from the transfer curve (Supplementary Fig. 3d). The

corresponding Fermi wave vector $k_f = (3\pi^2 n)^{1/3} \approx 0.04$ Å^{-1}, and the Fermi energy $E_f = \hbar v_f k_f$ is around 80 meV using an approximate Fermi velocity $v_f \sim 3 \times 10^5$ m/s. The large electron density and high Fermi level should largely come from the doping of defect states, since the intrinsic Fermi level should be located near the Dirac point for perfect crystals. Such high carrier density of defects is often caused by Cd site vacancies and impurity states in the growth process of Cd_3As_2 nanoplate. Supplementary Figure 3e gives the supercurrent density profile of junction 2 extracted via the Dynes and Fulton method, revealing the dominant role of bulk/surface states in supercurrent transport with finite edge contribution.

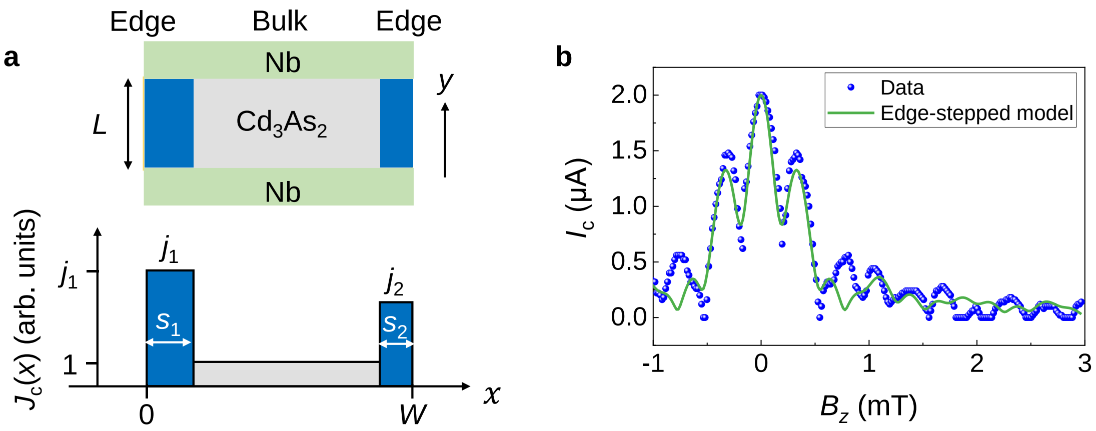

Supplementary Figure 4 | Edge-stepped nonuniform supercurrent model and fitting of $I_c(B)$ data for junction 1 at 10 mK.

a, Schematic of the edge-stepped supercurrent model. The upper panel depicts the top view of a Nb-Cd_3As_2 nanoplate-Nb junction. The lower panel describes the supercurrent distribution J_c along the *x* direction, where s_1 and s_2 denote the width of two edge channels, j_1 and j_2 corresponds to the normalized edge supercurrent density at two sides. L and W represent the channel length and width of the nanoplate junction, respectively.

b, Fitting of $I_c(B)$ data for junction 1 at 10 mK. The green curve is the fitting result based on the edge-stepped supercurrent model with $j_1 = 19.8$, $j_2 = 8.2$, $s_1 = 1.6$ μm and $s_2 = 1.3$ μm.

Here we consider a type of nonuniform supercurrent distribution as shown in Supplementary Fig. 4a. The normalized critical supercurrent density along the *x* direction $J_c(x)$ is given by

$$J_c(x) = \begin{cases} j_1 & (0 \le x \le s_1) \\ 1 & (s_1 < x < W - s_2), \\ j_2 & (W - s_2 \le x \le W) \end{cases}$$

where W is the junction width, s_1 and s_2 are the width of supercurrent edge channels, and j_1 and j_2 are the normalized amplitude of edge supercurrent density with respect to the bulk/surface supercurrent density as a unit of one. Generally, the magnetic field dependence on critical current $I_c(B_z)$ can be expressed by

$$I_c(B_z) = \left| \int_{-\infty}^{\infty} J_c(x) \exp\left(\frac{i2\pi L_{eff} B_z x}{\Phi_0}\right) dx \right|,$$

where $L_{eff} = L + 2\lambda$ is the effective junction length and satisfies the relation $L_{eff} W \Delta B_z = \Phi_0 = \frac{h}{2e}$. The experimental data for junction 1 can be fitted by the edge-stepped nonuniform supercurrent model, indicated by Supplementary Fig. 4b. The fitting results give the edge/bulk supercurrent ratio $j_1 = 19.8$ and $j_2 = 8.2$ at two sides, here assuming $j_1 \geq j_2$. The width of edge channels is fitted as $s_1 = 1.6$ μm and $s_2 = 1.3$ μm, which is close to the result obtained from the Dynes and Fulton method (Fig. 1f). The large value of j_1 and j_2 further confirms the dominance of edge supercurrent for junction 1 with a low bulk contribution.

Supplementary Note 1 | Derivation of the width and value of the edge supercurrent

Both the topological hinge states and non-Hermitian skin modes can form the supercurrent edge modes in junction 1. The topological hinge states are localized to the quasi-1D hinge, and the skin modes have a prominent spatial distribution near the sample edges. To clarify the origin of edge supercurrents, the supercurrent density profile $J_c(x)$ is fitted with the Gaussian function

$$J_c(x) = a_1 * \exp\left(-\frac{(x+b_1)^2}{c_1}\right) + a_2 * \exp\left(-\frac{(x-b_2)^2}{c_2}\right),$$

where a_1 and a_2 denote the peak height, b_1 and b_2 denote the peak position, and c_1 and c_2 determine the edge current width. For the $J_c(x)$ of junction 1 at 10 mK, as displayed in Fig. 1f, the Gaussian fitting gives the parameters $a_1 = 0.74$, $a_2 = 0.56$, $b_1 = 2.24$, $b_2 = 2.32$, $c_1 = 0.98$ and $c_2 = 0.89$. The width of supercurrent edge channels is defined as the full width at half maximum of the Gaussian peaks $\mathrm{FWHM} = 2\sqrt{c \cdot \ln 2}$, which takes values of 1.65 and $1.57\ \mu\mathrm{m}$ for the left and right edge channels, respectively. The observed supercurrent mediated by a single edge is $1.18\ \mu\mathrm{A}$ for the left edge and $0.8\ \mu\mathrm{A}$ for the right edge, both of which are much larger than the maximum supercurrent of a single hinge channel (~ 140 nA) in the short junction regime. The non-Hermitian skin modes provide an alternative channel for the edge supercurrent besides hinge channels. The width of the skin modes is approximately 1/4~1/3 of the system width in various non-Hermitian systems[2, 3, 4, 5, 6], in consistency with our observations here. Since the skin effect is sensitive to boundary conditions, asymmetric edge modes (in channel width and supercurrent amplitude) are observed in the nanoplate on two opposite sides. Figure 3c-d shows the supercurrent peak parameters varying with temperature, both of which are obtained from the $J_c(x)$ of junction 1 at different temperatures. As increasing temperature, the channel width and the supercurrent amplitude become gradually equivalent for the left and right edges, since the temperature increase would release the difference of skin modes, which are eliminated by the thermal fluctuations.

Supplementary Note 2 | Discussion on the origin of broadening edge channels

For the nanoplate junction with a long channel length (*e.g.*, junction 1), the edge-carried supercurrent is dominant with nearly vanishing bulk and surface contribution. It's found that the edge channel width exceeds 1.5 μm, much larger than the typical value of hinge channel width in Cd_3As_2 nanoplates. We attribute the broadening supercurrent edge channels to the mixture of non-Hermitian skin modes. In the following, we discuss the other mechanisms that may lead to a wide edge supercurrent channel.

The first possibility is involved with additional trivial edge modes, such as the dangling bond states and charge accumulation at the sample boundaries. Compared to the pure hinge-state scenario, the participation of trivial boundary modes would lead to the spatial broadening of edge channels. However, in our work, the topologically trivial conducting states can be safely ruled out based on the following reasons. First, from the current density profile (Fig. 1f), there are no bulk and surface states carried supercurrent. Due to the topologically protected nature of surface states, it is expected that the superconducting coherence length of surface states is larger than that of trivial states, that is, $\xi_{trivial} < \xi_{surface}$. Thus, the topologically trivial edge modes can hardly transmit supercurrents in a junction where the surface superconductivity has not been observed. Second, someone may say that the broadening edge channel arises from the boundary charge accumulation due to the band bending effect. But in the highly metallic Cd_3As_2 nanoplate studied here, the electrostatic screening effect is prominent, and the screening length is found smaller than 10 nm by taking the Fermi level and Fermi velocity into the Thomas-Fermi screening length formula. Such a short screening length cannot produce a 1.6 μm-wide charge accumulation layer. Last, the topologically trivial conducting states should be sample-dependent and wide or narrow trivial channels tend to occur randomly, however, the broadening edge channel is generally observed in our multiple devices. With the above issues taken into consideration, the topologically trivial conducting states can be ruled out as the main origin of broadening edge channels.

The second possible mechanism is related to the delocalization of hinge states. Bulk modes weakly coupled to hinge states can result in a wide edge supercurrent channel.

However, on the side wall of the nanoplate, the supercurrent carried by the two hinges with a distance of ~ 170 nm (thickness of the nanoplate) can still be distinguished (Supplementary Fig. 6b). Besides, in a previous device with 1 μm junction length (ref. 1), skin modes carried supercurrent is suppressed, while the hinge states carried supercurrent dominate with edge width < 600 nm, which is much smaller than that of the broadening edge supercurrent here in junction 1. This observation disagrees with the hinge state delocalization scenario since the hinge-bulk coupling should be independent of the junction length. Also, the Fermi wavelength of electrons in Cd_3As_2 is estimated to be $\lambda_f = \frac{2\pi}{k_f} \simeq 15$ nm (k_f is derived to be ~ 0.04 Å^{-1} from the carrier density), which limits the delocalization length. Therefore, the broadening supercurrent edge channel (~ 1.6 μm in width) cannot originate from the delocalization of hinge states.

References

1. Li, C.-Z. *et al.* Reducing Electronic Transport Dimension to Topological Hinge States by Increasing Geometry Size of Dirac Semimetal Josephson Junctions. *Phys. Rev. Lett.* **124**, 156601 (2020).
2. Lee, C. H., Li, L. & Gong, J. Hybrid Higher-Order Skin-Topological Modes in Nonreciprocal Systems. *Phys. Rev. Lett.* **123**, 016805 (2019).
3. Hofmann, T. *et al.* Reciprocal skin effect and its realization in a topolectrical circuit. *Phys. Rev. Res.* **2**, 023265 (2020).
4. Li, L., Lee, C. H. & Gong, J. Topological Switch for Non-Hermitian Skin Effect in Cold-Atom Systems with Loss. *Phys. Rev. Lett.* **124**, 250402 (2020).
5. Zhu, X. *et al.* Photonic non-Hermitian skin effect and non-Bloch bulk-boundary correspondence. *Phys. Rev. Res.* **2**, 013280 (2020).
6. Deng, K. & Flebus, B. Non-Hermitian skin effect in magnetic systems. *Phys. Rev. B* **105**, L180406 (2022).
